# Comparison of the use of internal limiting membrane flaps versus conventional ILM peeling on post-operative anatomical and visual outcomes in large macular holes

**DOI:** 10.1038/s41433-024-03024-1

**Published:** 2024-03-16

**Authors:** George Riding, Boon Lin Teh, David Yorston, David H. Steel

**Affiliations:** 1 Royal Free Hospital, Pond Street, London, UK; 2 Sunderland Eye Infirmary, Queen Alexandra Road, Sunderland, UK; 3 Gartnavel Hospital, Great Western Road, Glasgow, UK; 4 Bioscience Institute, Newcastle University, Newcastle Upon Tyne, NE1 3BZ UK

**Keywords:** Outcomes research, Retinal diseases

## Abstract

**Background:**

Idiopathic full-thickness macular hole (iFTMH) closure rates following conventional vitrectomy, gas tamponade and internal limiting membrane (ILM) peeling decrease when the minimum linear diameter (MLD) ≥ 500 microns. ILM flap creation has been proposed to improve closure in larger holes. This study evaluated the anatomical and functional impact of ILM flap introduction to routine practice in iFTMH ≥500 microns.

**Methods:**

Retrospective, interventional analysis of prospectively collected data of 191 eyes from consecutive surgeries for primary iFTMH ≥500 microns performed by two surgeons between June 2018 and June 2022, during which both surgeons replaced ILM peeling with ILM flap creation. Post-operative best-corrected visual acuity (BCVA) and anatomical closure were compared between Group 1 (ILM peel) and Group 2 (ILM flap) in an intention-to-treat analysis.

**Results:**

Rates of iFTMH closure were greater in the ILM flap group (77/80; 96.3%) than the ILM peel group (94/110; 85.5%) (OR = 4.37, 95% CI = 1.23–15.55, *p* = 0.023). A non-significant increase in post-operative BCVA improvement was observed in the ILM flap group (*p* = 0.084). There was no statistically significant difference in final BCVA (*p* = 0.83). Multivariate logistic regression found only MLD (OR = 0.993, 95% CI = 0.989–0.997, *p* = 0.001) and ILM flap group (OR = 5.795, 95% CI = 1.313–25.570, *p* = 0.020) predicted primary closure.

**Conclusion:**

ILM flap creation improves closure rates in larger holes and should be considered routinely in iFTMH ≥500 microns. Whether ILM flaps affect post-operative visual function remains uncertain.

## Introduction

Idiopathic full-thickness macular holes (iFTMH) are full thickness foveal defects [1]. They typically result in significant visual impairment and have an overall incidence of approximately 3–8 per 100,000 per annum, rising to 33 per 100,000 in women in their 60–70 s [2–5]. Since the introduction of internal limiting membrane (ILM) peeling in 1996 [6], the success rate of iFTMH anatomical closure following conventional surgery involving vitrectomy, ILM peeling and gas tamponade has climbed to around 96% overall in consecutive case series [7].

Macular hole size however is known to be an important factor in predicting surgical success, with closure reducing as size increases [8]. Whilst a minimum linear diameter (MLD) of 400 microns has been used as a cut-off for large full thickness macular holes [1], it has been shown that surgical success rate only starts to decline when macular hole width exceeds around 500 microns in size, to 90% or less in a large UK database study [7]. This was corroborated in another UK single centre series, which documented a further decline in closure above 630 microns [9]. Surgeons have therefore explored several surgical variations and adjuncts in these larger macular holes to improve success rates.

Michalewska et al. [10] first presented a novel technique of inverted ILM flap for the treatment of large macular holes, which appeared to improve closure rates. They hypothesised ILM flaps may stimulate gliosis and act as a scaffold for tissue proliferation. This was later supported by an experimental primate model which found activated Müller cells on the ILM flap producing neurotrophic factors that may contribute to macular hole closure [11]. Recently, randomised controlled studies (RCTs) have supported the benefit of ILM flaps in terms of closure in large holes [12, 13], whilst others have no found no conclusive benefit [14, 15]. A systematic review and meta-analysis has also shown a statistically significant improved closure rate of large macular holes with the use of ILM flaps compared to ILM peeling with an odds ratio of 3.95, but no difference in long-term visual outcomes [16]. The effect on visual acuity is unclear, although a large recent retrospective study has suggested a benefit [17].

There has been limited real-world data published on the routine use of ILM flaps in large macular holes over 500 microns, including those with variable symptom duration, which is also known to affect closure and visual outcomes, and which is typically constrained in randomised controlled trials [18]. The aim of this study was to evaluate the real-world anatomical and functional outcomes by comparing conventional ILM peeling to the use of ILM flaps in treating iFTMH of 500 microns or larger.

## Subjects and methods

We retrospectively reviewed all consecutive surgeries for primary iFTMH of 500 microns or larger, performed by two consultant vitreoretinal surgeons in two UK surgical units (DS in Sunderland Eye Infirmary and DY in Gartnavel Hospital, Glasgow). The patients were identified using prospectively collected data from the BEAVRS vitreoretinal database with a data collection period from June 2018 to June 2022. DS started using ILM flaps routinely in these cases from March 2019 and DY from October 2019.

Both surgeons used the same technique of ILM flaps, namely a superior single layer flap [19]. Prior to these dates all macular holes had been treated with vitrectomy, conventional ILM peeling with a peel radius of 1–2-disc diameters around the hole centre and predominantly long-acting gas tamponade (16–18% C2F6). Post-operatively, all patients were asked to posture face down for 1 day and avoid supine positioning for 1 week. Combined phacovitrectomy was performed in all phakic patients. Throughout the study period the surgeons’ techniques did not change in any other respects.

We excluded cases of vitrectomy performed with silicone oil tamponade, previous vitrectomised eyes, non-full thickness macular holes, persistent FTMH undergoing further re-operation and secondary macular holes including those which have developed in association with trauma, retinal detachment, myopia >6 dioptres or retinal dystrophies. Operations with less than 8 weeks follow-up were also not included in any post-operative best-corrected visual acuity (BCVA) analysis.

All iFTMH were imaged immediately preoperatively using spectral domain optical coherence tomography (SDOCT). The MLD was determined on the horizontal line scan where the hole was widest and measured manually using built-in-calliper tool [20]. Post-operatively, closure was assessed using SDOCT and specified as complete neurosensory retinal closure in any configuration without a retinal defect.

We collected baseline characteristics on age, sex, symptom duration, lens status, ocular co-morbidities, pre- and post-operative BCVA, pre-operative MLD, types of gas tamponade used and post-operative anatomical success. Primary outcome measures were post-operative BCVA; and successful anatomical closure following a single surgery.

We present descriptive data using tabular and graphical summaries. Statistical analyses were performed using MedCalc (MedCalc® Statistical Software version 20.218 (MedCalc Software Ltd, Ostend, Belgium; https://www.medcalc.org; 2023). Quantitative variables were presented as frequencies with percentages. Non-parametric continuous variables were reported as median, IQR, and range. Differences in median values were assessed with Mann-Whitney tests. We expressed results using odds ratios (ORs) and their 95% confidence intervals (CIs). Multivariate analysis by stepwise logistic regression was used to examine variables associated with primary closure of iFTMH.

We included all consecutive eyes in the analyses dependant on their follow-up duration. Eyes had to have had at least 2 weeks post-operative follow up to be included in the anatomical analysis and 8 weeks to be included in the visual outcome analysis, Supplementary Fig. 1. We included all eyes with attempted ILM flap creation in an intention-to-treat (ITT) analysis.

## Results

191 consecutive eyes with holes >500 microns, operated on by the two surgeons within the specified time period were identified from the BEAVRS database, with 111 eyes undergoing ILM peeling in the first time period (ILM peel group) and 80 eyes undergoing ILM flap in the second time period (ILM flap group). Two ILM flaps were avulsed in group 2 but were included in the intention-to treat analysis.

The baseline characteristics of the two treatment cohorts are shown in Table 1. No statistically significant differences were observed in the age, sex, MLD, BD, baseline phakic status, gas tamponade used, or duration of follow-up between the two groups. The symptom duration prior to surgery was significantly greater in the ILM flap group (median 10 months) than the ILM peeling group (median 7 months, *p* = 0.001), whilst the pre-operative BCVA was also worse in the ILM flap group than the ILM peeling group (1.1 to 1.0 logMAR, *p* = 0.015).

**Table 1:** Baseline variables of the two groups.

	ILM peel group 111 eyes		ILM flap group 80 eyes		*p* value
Age, years (median, IQR, min–max)	70, 65–75, 48–87		71, 66–75, 48–87		0.51
Female sex, *n* (%)	99 (89.2)		70 (87.5)		0.72
Symptom duration, months (median, IQR, min–max)	7, 5–10, 2–40		10, 6–14, 1–38		0.001
MLD, microns (median, IQR, min–max)	595, 554–657, 502–875		592, 547–670, 508–1232		0.98
BD, microns (median, IQR, min–max)	1086, 968–1244, 701–1836		1150, 1011–1290, 693–1582		0.14
Baseline VA, logMAR (median, IQR, min–max)	1.0, 0.85–1.20, 0.58–1.98		1.1, 0.95–1.40, 0.50–1.98		0.015
Pseudophakic (*n*, %)	17 (15.3)		11 (13.8)		0.76
Tamponade	C2F6	108 (97.3)	C2F6	80 (100.0)	0.27
	SF6	3 (2.7)	SF6	0 (0.0)	
Follow-up, days (median, IQR, min–max)	139, 110–213, 38–575		157, 99–221, 17–514		0.88

*ILM* internal limiting membrane, *MLD* minimum linear diameter, *BD* base diameter, *VA* visual acuity, *C2F6* hexafluoroethane, *C3F8* perfluoropropane.

The ILM peel and the ILM flap groups had 110 (99.1%) and 80 (100%) eyes eligible for anatomical closure assessment with follow-up >2 weeks post-op, respectively, whilst 107 (96.4%) and 72 (90%) eyes of the ILM peel and ILM flap group respectively were eligible for visual function assessment, with acuity data at >8 weeks post-op.

Post-operative outcomes are shown in Table 2. FTMH closure was more likely in the ILM flap group, with 77 (96.3%) in the ILM flap group closing, compared to 94 (85.5%) of the ILM peel group (OR = 4.37, *p* = 0.023). Of the 19 eyes without primary closure, all but 1 had revision surgery with 15 of those 18 achieving closure. The final closure rate was therefore 186 of the 190 (97.9%) eyes in total. An increase in post-operative BCVA improvement was observed in the ILM flap group (median 0.60) compared to the ILM peel cohort (median 0.52, *p* = 0.084). There was no difference in final BCVA, or in the proportion gaining or losing ≥0.2 logMAR between the two cohorts. The results were unchanged when considering only those with primary closure.

**Table 2:** Anatomical and visual outcomes.

	ILM peel group	ILM flap group	*p* value
Closure (*n*, %)	94/110 (85.5%)	77/80 (96.3%)	0.023*
Final VA, logMAR (Median, IQR, min–max)	0.50, 0.32–0.78, 0.0–1.98	0.56, 0.40–0.70, 0.10–1.98	0.83
VA improvement, logMAR (Median, IQR, min–max)	0.52, 0.30–0.70, 0.09–1.48	0.60, 0.35–0.83, 0.20–1.58	0.08
VA loss by 0.2 logMAR or more (*n*, %)	5 (4.7%)	1 (1.4%)	0.40
VA improvement by 0.2 logMAR or more (*n*, %)	95 (88.8%)	67 (93.1%)	0.44

*Odds ratio = 4.37, 95% confidence limits 1.23–15.55, *p* = 0.023.

*ILM* internal limiting membrane, *VA* visual acuity.

Multivariate logistic regression showed that only two factors predicted primary closure, namely MLD and being in the ILM flap group, Table 3.

**Table 3:** Multivariate logistic regression for anatomical closure.

	Co-efficient	Odds ratio	95% confidence interval	*p*
Constant	6.362			<0.001
MLD (μm)	−0.007	0.993	0.989–0.997	0.001
Group 2 (ILM flap)	1.757	5.795	1.313–25.570	0.020

*MLD* minimum linear diameter, *ILM* internal limiting membrane.

## Discussion

Using prospectively collected real-world data from two surgeons before and after the routine use of ILM flaps in iFTMH > 500 microns in size, we show that the introduction of ILM flaps improved the closure rate with both clinical and statistical significance. The closure rate increased from 86 to 96%. Multivariate analysis showed that ILM flap use had a beneficial effect on closure with an OR of 5.8.

As mentioned in the introduction, recently published real-world based studies have proposed that the definition of a large FTMH, when the MLD is >400 microns, is not a pragmatic demarcation point in terms of surgical success [1, 7, 10, 21]. 500 microns may be a more appropriate threshold where the success rate drops from above 95%, to below 90%. We observed closure rates in FTMH > 500 microns improving from 86 to 96% following the introduction of ILM flaps. This closure rate is similar to that observed in smaller holes, following conventional surgery [7, 9].

Our study corroborates previous retrospective, non-randomised studies investigating the impact of ILM flap on closure rates in large FTMH: Baumann et al. [22]. observed a similar closure rate in their study of 117 eyes with an MLD > 400 microns, with 99% achieving successful closure in the ILM flap group compared to 88% in ILM peel group. Rizzo et al. [17]. in their comparative study of over 620 eyes also observed a significant improvement in closure rates from 79 to 92% in all holes, whilst specifically in holes >400 microns, an increase from 79% closure with ILM peeling to 96% with ILM flaps was observed. A meta-analysis of 4 RCTs and 4 retrospective studies conducted by Shen et al. [16]. also reported superior closure rates with ILM flaps, although they all included holes >400 microns rather than 500 microns. Our unadjusted OR of 4.4 concurs with their pooled OR of 4, whilst our multivariate analysis suggests that in larger holes (>500 microns) the effect is greater still.

Despite the higher closure rate with ILM flaps, similar post-operative visual function was observed in both groups. The change from ILM peel to ILM flap was instituted by both surgeons just before the coronavirus (COVID-19) pandemic, and the ILM flap group therefore included patients with significantly longer symptom durations and worse baseline visual acuities than the ILM peel group as other procedures were prioritised in the UK during phases of the pandemic [23]. Both variables are established negative predictors of post-operative visual function and indeed closure, potentially confounding any beneficial effect on post-operative visual function [16]. Furthermore, all patients with recalcitrant holes in our study underwent repeat surgery with high closure rates, further reducing the difference in acuity at their last visit. Despite these observations it is worth noting that we showed greater improvement in visual acuity in the ILM flap group, although there is a possibility that this difference occurred by chance.

Whether ILM flaps improve post-operative visual function is disputed in the current literature. Of the 8 RCTs published to date comparing ILM flap use to conventional ILM peeling (Table 4), only 3 report significantly improved post-operative visual function in the ILM flap group, albeit these RCTs were conducted in large holes and had the largest sample sizes [10, 12, 13]. Of the 4 RCTs demonstrating no significant improvement in post-operative visual function, 3 were conducted in small-medium holes, whilst the other on larger holes which did not reach significance had a smaller sample size than the other large hole RCTs [14, 21, 24, 25]. Furthermore, Ventre et al. [21]. in their RCT of small-medium holes further demonstrated worse final post-operative macular sensitivity following ILM flap than ILM peel. There is also contradictory evidence from published retrospective studies: Rizzo et al. [17] observed an improvement in post-operative visual function in all sized holes, including those >400 microns, whilst Baumann et al. [22] and Yamashita et al. [26] did not observe a difference in holes >400 microns. The data to date suggests that the main benefit of ILM flaps is observed in patients with larger holes, where the risk benefit ratio of the more prolonged surgery required to create a flap is clearer.

**Table 4:** Published randomised controlled trials investigating ILM flap technique versus conventional ILM peeling in macular hole surgery.

Study	MLD inclusion criteria	Group	No of eyes	MLD (μm)	Closure rate (%)	*p* value	Preop VA (logMAR)	Post-op VA (logMAR)	p value	Follow-up (months)
Michalewska et al. [10]	>400 μm	ILM peeling	51	698	88	0.01	0.92	0.77	0.001	12
		ILM flap	50	759	98		1.11	0.55		12
Manasa et al. [13]	≥600 μm	ILM peeling	48	658	88	0.40	1.10	0.86	0.010	3
		ILM flap	43	673	95		0.99	0.67		3
Kannan et al. [14]	≥600 μm	ILM peeling	30	760	77	0.17	0.79	0.65	0.060	6
		ILM flap	30	803	90		0.75	0.53		6
Velez-Montoya et al. [15]	>400 μm	ILM peeling	12	522	92	0.85	0.93	0.71	Not stated	3
		ILM flap	12	609	92		0.95	0.62		3
Ehrhardt et al. [25]	>250 μm	ILM peeling	30	375	93	1	0.70	0.20	0.450	3
		ILM flap	30	405	93		0.70	0.20		3
Agrawal et al. [12]	>600 μm	ILM peeling	75	750	93	<0.001	1.02	0.31	0.001	12
		ILM flap	75	766	100		1.03	0.19		12
Leisser et al. [24]	<400 μm	ILM peeling	9	244	100	1	1.30	0.94	0.700	3
		ILM flap	7	275	100		1.34	0.90		3
Ventre et al. [21]	≤400 μm	ILM peeling	25	254	100	1	0.72	0.19	0.398	12
		ILM flap	25	269	100		0.76	0.22		12

Recent evidence published by Chou et al. [27] in FTMH < 400 microns treated with ILM flaps shows significantly improved post-op BCVA at 1, 3 and 6 months, but not at 12 months. This is in keeping with the findings of Kwak et al. [28] who observed superior post-operative BCVA at 1 month post-op, but not at 3-, 6- or 12 months, where propensity-score matching was used to eliminate the influence of important baseline confounders such as MLD and pre-operative BCVA. The meta-analysis by Shen et al. [16] also reported an improved post-operative BCVA at 3-months, but not 6 months. We did not analyse post-operative visual function at multiple time intervals and required at least 8 weeks follow-up for visual outcome analysis, therefore we are unable to compare our findings to the short-term improvements in visual function reported. Notably, in our study the follow-up in the two groups was comparable. It is possible that the early improvement in visual function noted by these authors arises from the superior initial closure rates, and at later time points after revision surgery in non-closed holes the effect diminishes. It has also been hypothesised that ILM flaps may mechanically obstruct the restoration of the ellipsoid zone and external limiting membrane, essential to good post-operative visual function in FTMH repair [29–32]. Future studies with larger cohorts and long follow-up of at least 12 months are required to evaluate whether the initial impact of increased primary closure is accompanied by any beneficial or detrimental effect on post-operative visual function.

Since the conception of the ILM flap technique by Michalewska et al. [10], multiple variations have been proposed which can be broadly divided into single layer covering techniques and flap insertion techniques as originally described. There is no definitive evidence published that one technique is superior to the other, although one study suggests single layer techniques have improved visual results in the short term [33]. In our study, both surgeons used the large superior semicircular inverted ILM flap technique described by Chen et al. [19], a variation of the covering single layer flap, and therefore we avoided any potential negative effects of the insertion technique.

Unlike randomised trials, our patients were consecutive cases, without exclusions for prolonged duration or co-morbidity. These results are likely to be applicable to the real-world experience of retinal surgeons. Our study is however not without limitations, notably a lack of randomisation meaning that we cannot discount unrecognised confounding effects, although this was mitigated as our groups were consecutive cases, and with prospective data collection. Both surgeons had over 15 years of experience of ILM peeling for FTMH prior to the study start date making experiential effects unlikely. Similarly, the surgery was standardised between both groups including for post-operative positioning. Both surgeons only asked patients to posture for 24 h whilst they were inpatients, making any confounding effect of posturing also improbable. Our population was predominantly White British, with a relatively long symptom duration limiting generalisability in different populations. Despite these limitations, we feel the results from our study can be translated to routine clinical practice for all FTMH > 500 microns as our data come from the consecutive cases of two surgeons and analysed as an ITT analysis accepting that 2 ILM flaps avulsed during creation, acknowledging the technical challenges of performing the procedure. Finally, we did not describe the exact closure pattern, but it should be noted that our definition of closure was type 1 without a neurosensory defect, and type 2 closures were classed a non-closure.

In conclusion in this consecutive real world by two independent surgeons case series we have demonstrated that the use of ILM flaps in large FTMH > 500 microns improves closure rates. Whether ILM flaps effect post-operative visual function remains unclear and requires further study. The use of ILM flaps should be considered routinely in patients with iFTMH and a MLD of greater than 500 microns.

## Summary

### What was known before

- The use of ILM flaps probably improves FTMH closure in holes greater than 400 microns.

### What this study adds

- Robust, generalisable, real-world evidence that the use of ILM flaps as part of the routine surgical management of FTMH > 500 microns will substantially increase the likelihood of closure and reduce the  need for re-operation.

### Supplementary information

Supplementary Figure 1

## Data Availability

The data that support the findings of this study are available from the corresponding author upon reasonable request.
