# Supplementary figures and images for: Comparison of the use of internal limiting membrane flaps versus conventional ILM peeling on post-operative anatomical and visual outcomes in large macular holes
Source: Eye (Lond). 2024 Mar 16;38(10):1876–81. doi: 10.1038/s41433-024-03024-1 (PMC11226651; doi:10.1038/s41433-024-03024-1)

SUPPLEMENTARY FIGURE 1; Flow diagram for analysis eligibility

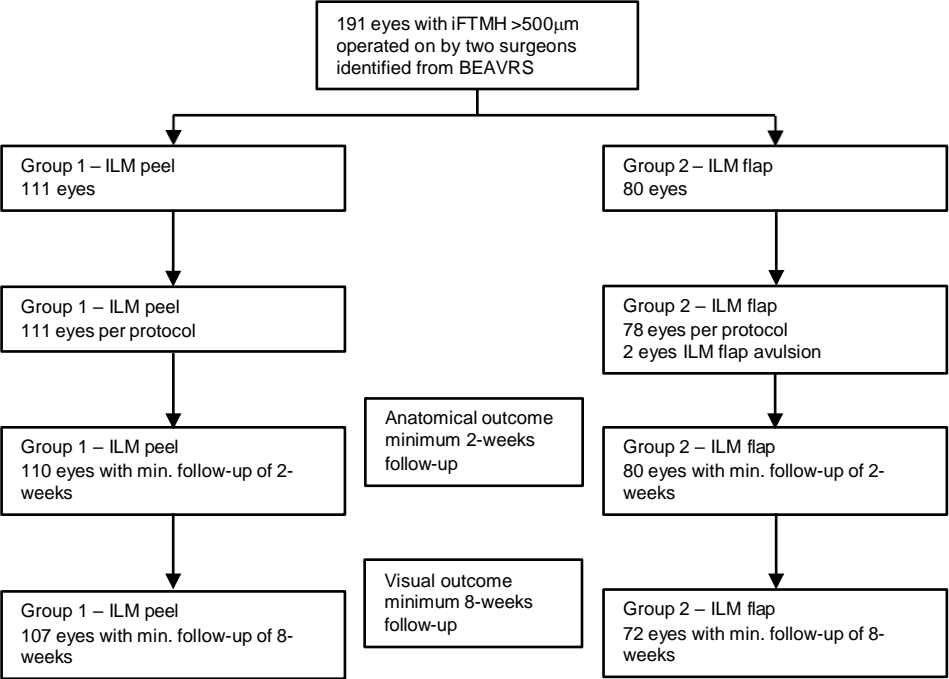

Supplement: Supplementary file 1 — Supplementary Figure 1 [file 41433_2024_3024_MOESM1_ESM.pdf]
